# Modeling Long-Term Erythropoietic Recovery After Allogeneic Stem Cell Transplants in Pediatric Patients

**DOI:** 10.3389/fped.2020.584156

**Published:** 2020-11-30

**Authors:** Erik G. J. von Asmuth, Alexander B. Mohseny, Hein Putter, Marco W. Schilham, Arjan C. Lankester

**Affiliations:** ^1^Willem Alexander Children's Hospital, Leiden University Medical Center, Leiden, Netherlands; ^2^Department of Medical Statistics, Leiden University Medical Centre, Leiden, Netherlands

**Keywords:** erythropoiesis, retrospective, pediatric stem cell transplantation, leukemia, blood group (AB0)

## Abstract

Long term erythropoietic reconstitution after allogeneic hematopoietic stem cell transplantation (alloHSCT) has not been extensively studied. We aimed to describe erythropoietic reconstitution as an indicator of long-term graft function by modeling hemoglobin levels during the first 3 years post HSCT in pediatric patients. We retrospectively included 414 patients and 11,957 measurements. The largest hemoglobin increase was at day 45 and levels reached a steady state at day 648 with a level of 7.48 mmol/L. In patients transplanted for hematological malignancies hemoglobin levels normalized faster (*p* < 0.0001). Increasing patient age correlated with faster recovery (*p* < 0.0001), while donor age had no influence. Conditioning, donor type and graft source did not influence recovery significantly. In the ABO mismatched group there was a transient negative effect on hemoglobin levels, and a delay in reticulocyte recovery (21 vs. 19 days; *p* = 0.012). In contrast, hemoglobin levels reached a higher plateau beyond 9 months in these patients (*p* < 0.0001). After alloHSCT, experiencing a CMV reactivation negatively affected reconstitution (*p* = 0.034), while EBV reactivations and acute graft vs. host disease did not. In summary, erythropoietic recovery was mainly influenced by patient factors and primary disease, and less influenced by donor factors.

## Introduction

Allogeneic stem cell transplantation (alloHSCT) is an invasive procedure, aiming to replace the diseased patient's hematopoietic system and to provide a long-term healthy hematopoietic system. When investigating the hematopoietic system after alloHSCT, studies mainly focused on describing leukocyte populations and functioning whereas studies investigating erythropoiesis focus on short term outcome, while long-term erythropoiesis is left unexplored (1–3).

The impact of conditioning regimens on erythropoietic recovery after alloHSCT has been evaluated in several studies but remains unresolved. A reduced intensity conditioning (RIC) regimen has been associated with less red blood cell transfusions compared to a myeloablative regimen in one study (2) while another study reported delayed reticulocyte recovery (4). A third study reported no difference in erythropoietic recovery between reduced and myeloablative conditioning (1).

Regarding graft source, one study in adults showed slower reticulocyte recovery in patients transplanted with bone marrow vs. peripheral blood stem cells. The effect was independent of ABO match status. However, the long-term effect of graft source on erythropoietic recovery has been left largely unexplored (1).

ABO mismatches in alloHSCT are subdivided into minor and major mismatches. Major mismatch, indicating that circulating antibodies are present in the patient against a donor blood group antigen (5), has been associated with pure red cell aplasia (PRCA), haemolysis, lower reticulocyte counts, a higher number of blood transfusions required post alloHSCT, and delayed erythropoietic engraftment (5–11). Minor ABO mismatch, implying that donors have circulating antibodies against a patient blood group, have been associated with haemolysis post alloHSCT, but have a smaller clinical impact (5).

Studies regarding ABO mismatch have mainly reported data in adult patients. The few studies focussing on pediatric alloHSCT patients report inconsistent findings. A single-center study in children only describes one patient with PRCA out of 55 patients with a major ABO mismatch and no impact on survival or Graft vs. Host disease (GvHD). An EBMT registry study in children with hemoglobinopathies transplanted with a matched sibling donor reported similar findings (12). This indicates that the effect of an ABO mismatch might be different in children compared to adults.

While most studies on erythropoietic recovery have focussed on the short-term effects, long term effects are increasingly more relevant with recent improvements in survival. The question how erythroid haematopoiesis develops after the first few months and which donor, patient and transplantation characteristics influence long lasting haematopoiesis is largely unexplored. Therefore, we developed a mixed model to describe hemoglobin levels in children during the first 3 years post SCT and evaluated the influence of host characteristics such as age, gender and underlying disease, donor factors such as age and relation to the patient, and transplantation related variables.

## Methods and Materials

### Ethics

Data collection was approved by MREC Leiden The Hague Delft. Written informed consent was obtained from all subjects or their legal guardians. As this study was non-interventional in nature, it was reviewed and approved by the local scientific committee and did not require additional approval of a regional medical ethics committee. Patient data was handled in compliance with the declaration of Helsinki and local laws and regulations.

### Study Design

Pediatric patients treated with alloHSCT between the first of July 2004 and the first of July 2018 at the Leiden University Medical Center were retrospectively included in the study. Pediatric patients transplanted for hematological malignancies, severe aplastic anemia, inherited bone marrow failure syndromes and inborn errors of immunity were included. Only first transplants were included. Patients receiving combined grafts were excluded.

For all patients, baseline and donor characteristics were retrieved, and survival and relapse data were collected. Next, all hemoglobin and reticulocyte measurements, up to 3 years post alloHSCT, were included for analysis.

All measurements after a subsequent transplant or after relapse were excluded. Hemoglobin measurements before day 30 were excluded to avoid impact of transfusions during conditioning-related aplasia. To avoid major complications such as infection and graft vs. host disease and their treatment having an impact, measurements during a hospital readmission after 60 days post HSCT were excluded.

Hemoglobin levels were modeled using a mixed model. Differences in mean levels after 1 year were tested using ANOVA and *T*-test. Exact modeling approach and *P*-value calculation can be found in Supplemental Text 1.

We did not incorporate transfusions in the model, as we aimed to investigate long-term effects, and only 11 patients required transfusions between year 1 and 3. Diagnoses for these patients were beta-thalassemia (*N* = 6) malignancies (*N* = 3) and sickle cell disease (*N* = 2). All transfusions were related to either relapse or secondary graft failure.

## Results

A total of 414 patients were included and a total of 11,957 hemoglobin measurements were performed within the study interval. On average, 29 measurements per patient were included for the analyses. Details on patients and samples are available in Table 1.

**Table 1:** Patient and sample characteristics.

Patient age (median, range)	8.6 years (0.2–18.6)
Donor age (CB excluded)	27.2 years (1.1–67.6)
Patient sex	
Male	266
Female	148
Donor sex	
Male	222
Female	192
Stem cell source	
Bone marrow (BM)	301
Peripheral blood stem cells (PBSC)	68
Cord blood (CB)	45
Donor type	
Unrelated donor	248
HLA identical sibling	120
Other related donor	46
ABO blood group matching	
Matched	217
Minor mismatch	92
Major mismatch	79
Bidirectional mismatch	26
Diagnosis	
Hematological malignancy	209
Non-immunological hematopoietic disorders	136
Haemoglobinopathies	76
Severe aplastic anemia	28
Other hematopoietic disorders	32
Inborn errors of immunity	69
SCID	20
Non-SCID	49
Days between sample and alloHSCT (median, range)	93 days (30–1,094)

As a starting point, a model was defined using only time as a dependent variable. In this model, the effect of time post alloHSCT on hemoglobin levels was highly significant (*p* < 0.0001, *R*^2^ = 0.259).

**Figure 1:**
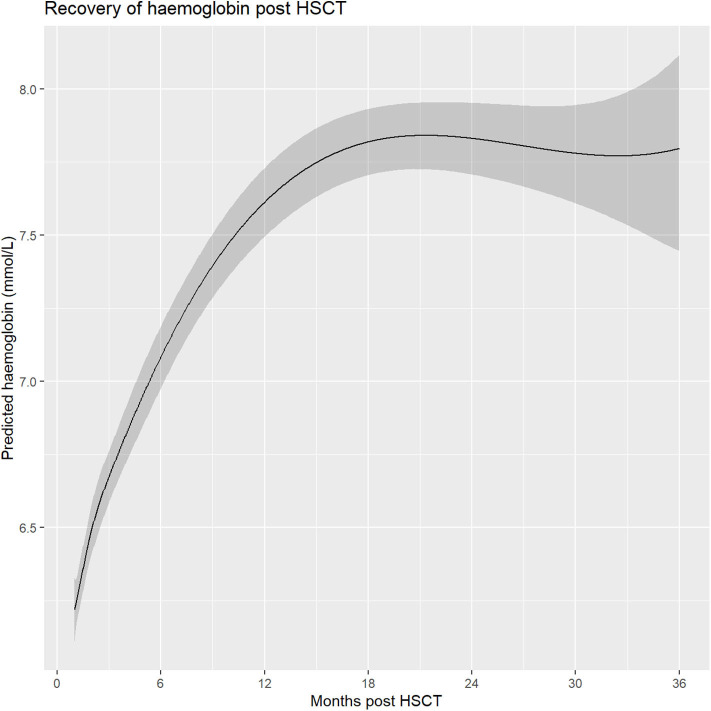
Overall hemoglobin recovery based on a mixed model.

Initially, recovery rate increased, up to a maximum increase of 0.01 mmol/L per day at day 45 (Figure 1). Afterwards there was a period of stable recovery, tapering off around day 500 and reaching its maximum at day 648 at a hemoglobin level of 7.84 mmol/L (95% CI 7.73–7.96 mmol/L). As an alternative analysis of the data we averaged the values for each patient over subsequent intervals of 50 days, starting from day 30. This resulted in a similar recovery profile as obtained using the mixed model, but with less stable means and confidence intervals, showing that the model predictions were in line with the observed values (Supplementary Figure 1).

During follow-up, 2 patients had suspected pure red cell aplasia. Neither of them underwent a bone marrow biopsy to confirm the diagnosis, and both reconstituted reticulocytes to levels above 30 × 10^9^/l within 2 months post alloHSCT. None of the patients received erythropoietin or Romiprostim, but two patients received Eltrombopag to boost engraftment. Manual inspection of the hemoglobin levels did not reveal an effect of Eltrombopag in these patients.

**Figure 2:**
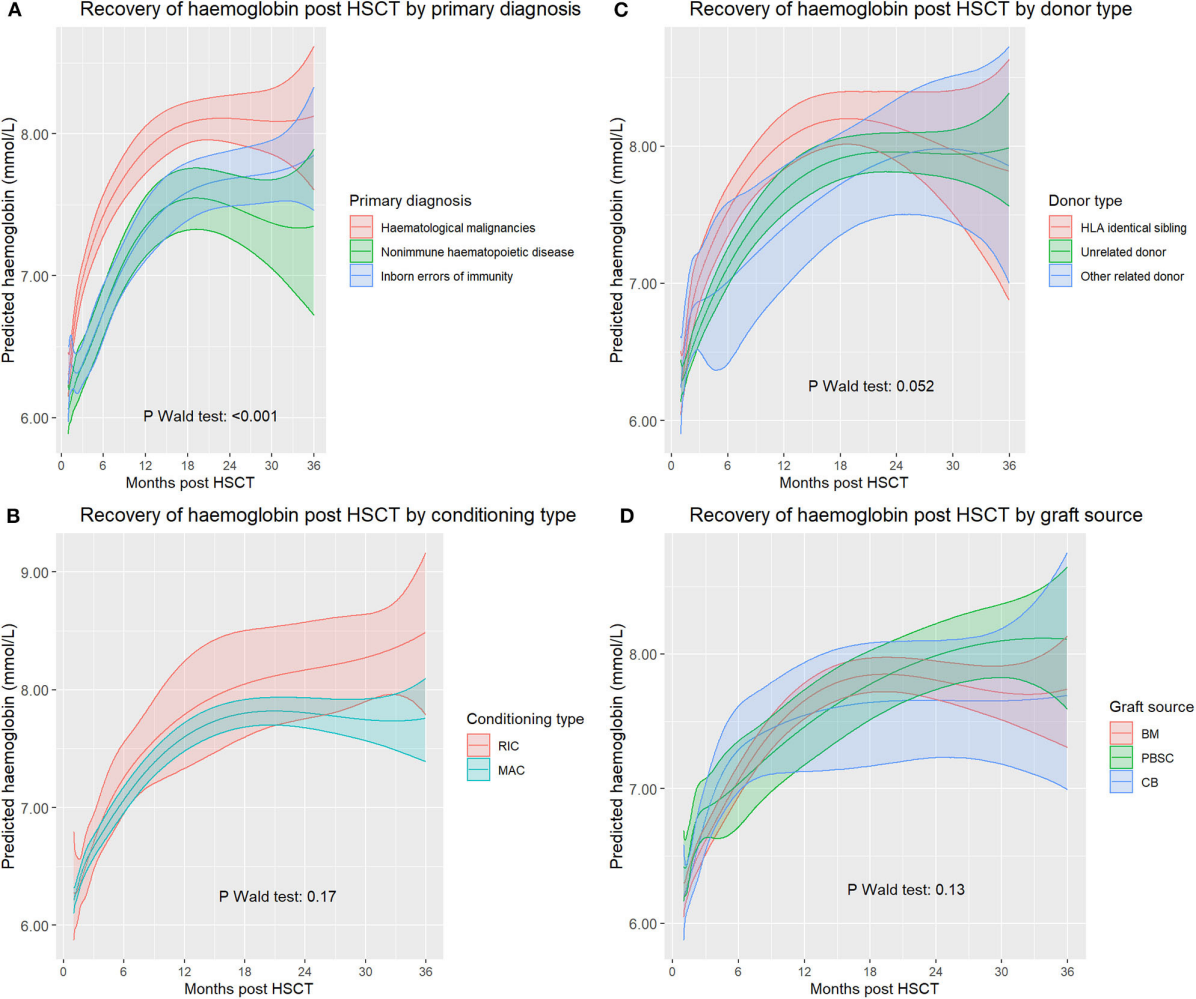
**(A)** Haemoglobin recovery split by diagnosis, **(B)** Haemoglobin recovery split by myeloablative conditioning (MAC) vs. reduced intensity conditining (RIC), **(C)** Haemoglobin recovery split by donor type, with thallasemia and sickle-cell anemia excluded. **(D)** Haemoglobin recovery split by graft source. BM, Bone marrow; CB, Cord blood; PBSC, Peripheral blood stem cells.

Primary diagnosis of HSCT recipients was significantly associated with hemoglobin recovery (*p* < 0.0001, Figure 2A). Patients with hematopoietic malignancy as primary diagnosis recovered faster and had a significantly higher hemoglobin level between day 44 and day 744 than patients transplanted for inborn errors of immunity. There was no significant difference in predicted hemoglobin level between patients transplanted for inborn errors of immunity and patients transplanted for non-immune haematopoietic disorders.

We also averaged actual hemoglobin values in the time interval between 1 and 3 years after HSCT per patient, and then compared these averages between diagnoses. The average values were significantly influenced by primary diagnosis (ANOVA *p* < 0.0001). Patients with hematological malignancies had a higher hemoglobin value compared to the other groups (8.1 vs. 7.5 mmol/L, 95% CI 0.75–0.35, *p* < 0.0001), patients transplanted for non-immune haematopoietic disorders and immune disorders both had a lower hemoglobin value compared to all other diagnoses (7.6 vs. 7.9, *p* = 0.002, and 7.5 vs. 7.9, *p* = 0.002, respectively).

We investigated the effect of the conditioning regimen on Hb levels by comparing reduced intensity conditioning (RIC) vs. myeloablative conditioning (MAC). The influence of conditioning in the mixed model was not significant (*p* = 0.17, Figure 2B), and there was no significant difference between RIC and MAC in average hemoglobin level per patient 1 year after HSCT (*p* = 0.11).

To analyse the effect of donor type, patients transplanted for thalassemia and sickle cell anemia were excluded from this analysis as related donors could be carriers of thalassemia or sickle cell anemia, which might therefore create a bias when comparing related vs. unrelated donors (76 patients excluded, 31 identical sibling donors, 31 unrelated donors, 14 haploidentical donors). The overall effect of donor type on hemoglobin recovery was only borderline significant (*p* = 0.052).

Identical sibling transplants show faster initial hemoglobin recovery than patients transplanted with matched unrelated donors (Figure 2C). Haploidentical transplants did not differ significantly from either category but were limited in numbers. One year after alloHSCT, there was no significant effect of donor type on average hemoglobin values (*p* = 0.38).

The overall effect of graft source on hemoglobin recovery was not significant (*p* = 0.13, Figure 2D). Initial recovery for PBSC appeared slightly faster, but this was not significant. CB transplants did not seem to have a different recovery than BM transplants. Average hemoglobin levels between 1- and 3-years post stem cell transplant were not significantly different between donor types (*p* = 0.75).

**Figure 3:**
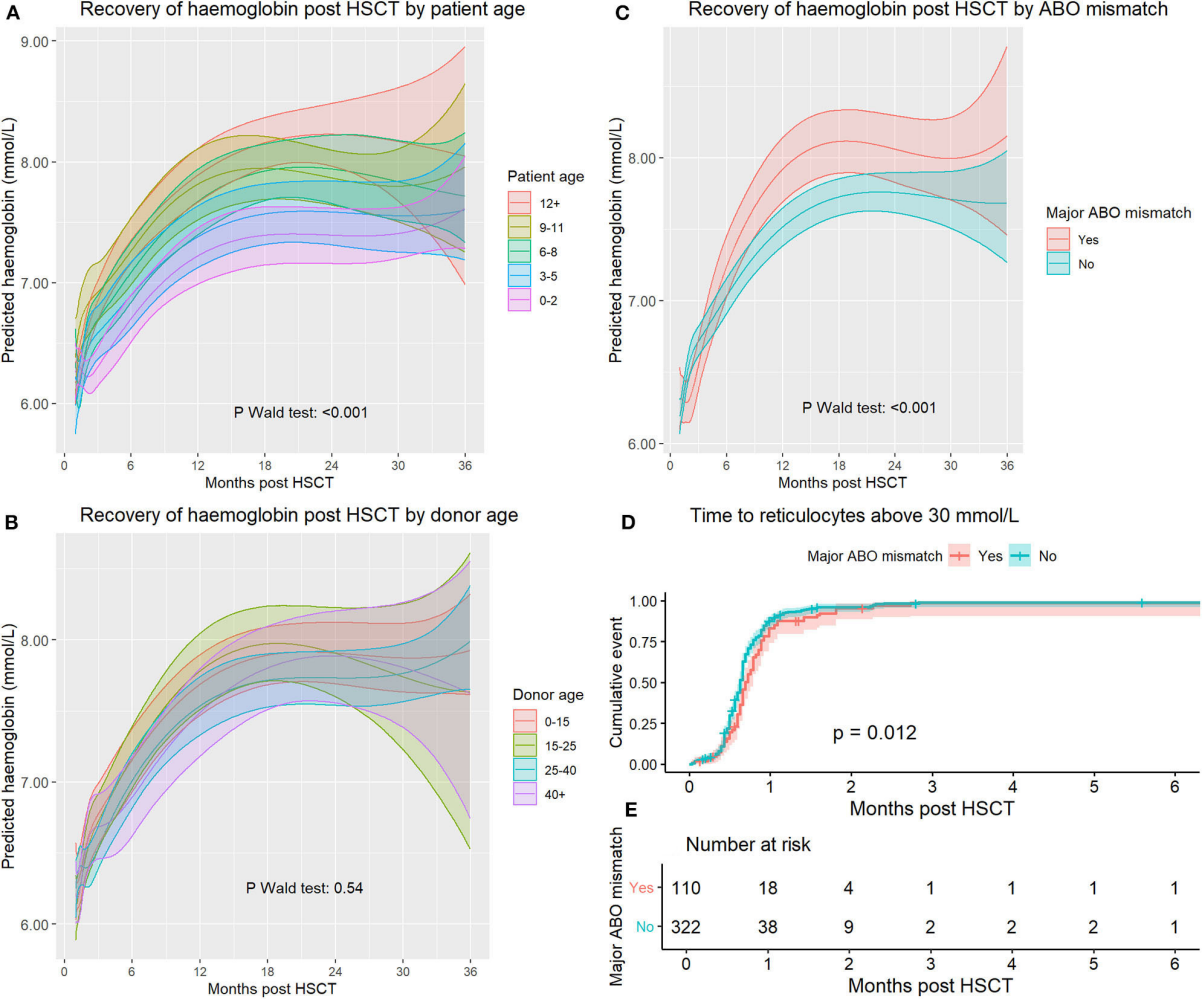
**(A)** Haemoglobin recovery split by patient age at time of transplant. **(B)** Haemoglobin recovery split by donor age. **(C)** Haemoglobin recovery split by a major ABO mismatch. **(D)** Time to reticulocyte recovery split by a major ABO mismatch. **(E)** Number at risk for **(D)**.

The influence of patient age was analyzed by categorizing age in 3-year groups. The influence of patient age on hemoglobin recovery was significant (*p* < 0.0001, Figure 3A). A higher patient age was associated with higher hemoglobin levels during the entire period of 3 years after transplantation.

To analyse the effect of donor age, we categorized donor age using the following categories: <15, 15–25, 25–40, and 40+ years of age. Cord blood transplants were excluded from this analysis. The overall influence of donor age on hemoglobin recovery was not significant (*p* = 0.54). After analyzing model-based predictions, there were no significant differences between any groups at any timepoint (Figure 3B). Using reference values for Hb levels matched for patient age and sex resulted in a closer approximation of actual levels than using reference values corresponding to donor age and sex (Supplemental Figure 2).

The effect of a major ABO mismatch on hemoglobin recovery was significant (*p* < 0.0001, Figure 3C). Initially, there was a lower hemoglobin level in the major mismatch group, but this reversed at day 121, and between day 263 and day 629, hemoglobin reached higher levels in the major ABO mismatch group. At 1 year after transplantation average hemoglobin levels were significantly higher in patients with an ABO mismatch (*T*-test *p* = 0.0036, CI 0.12–0.61). Analyzing major, minor and bidirectional mismatches separately did not lead to improved model predictions and are not reported.

To assess the influence of an ABO mismatch on erythropoietic recovery shortly after SCT, we analyzed reticulocyte recovery because this is not affected by transfusions but is delayed in pure red cell aplasia. We used survival analysis and measured time to first reticulocyte count above 30 × 10^9^/l (Figure 3D, p log-rank = 0.012). Median recovery time for the mismatched and non-mismatched groups was 21 and 19 days, respectively.

When analyzing the predictive accuracy of the models, the model including only time had an *R*^2^ of 0.259. Adding diagnosis to this model led to the most substantial improvement with a coefficient of partial determination of 3.6%. Detailed analysis of the predictive accuracy of all models is provided in Supplemental Table 1 and the associated text.

To investigate the effects of acute GvHD, CMV and EBV reactivation on long-term hemoglobin levels, we analyzed the data, and excluded measurements from the first 100 days from our cohort to avoid the direct effects of these events.

Acute GvHD and EBV reactivation did not have an influence on long-term hemoglobin levels (*p* = 0.81 and *p* = 0.36 respectively). For patients that had encountered a CMV reactivation, predicted hemoglobin levels were significantly lower only between the start of the analysis period (100 days) and 6 months, but had there was no effect on long-term erythropoiesis (*p* = 0.034, Supplemental Figure 3).

## Discussion

In this study, we investigated long-term erythropoietic recovery after alloHSCT in pediatric patients and factors influencing hemoglobin recovery. We found a strong influence of primary disease, leading to fast recovery in patients transplanted for malignancies, and an effect of patient age but not donor age. While there was a transient negative effect of a major ABO mismatch shortly after transplant, this effect remarkably reversed within 9 months. Stem cell source and donor type did not appear to have an influence. These findings indicate that patient factors are the main determinants of long term erythropoietic recovery as opposed to hematopoietic stem cell age or other donor related factors.

When studying myeloablative vs. reduced intensity conditioning, existing studies provide conflicting evidence (1, 2, 4). In our study, we did not find convincing evidence for a long-term difference between these approaches.

In our cohort, the use of erythropoietic growth factors was negligible. Erythropoietin has been shown to reduce transfusion requirements and speed up erythropoietic recovery, both in the adult and pediatric population (13–15). Recently, Eltrombopag has shown good results stimulating erythropoiesis in SAA patients treated with immunotherapy (16), a strategy that might be considered in stem cell transplant patients suffering from inadequate erythropoiesis.

Current practice in donor selection hierarchy is to avoid ABO mismatches between donor and patient. In accordance with clinical experience, we found a slight delay in reticulocyte recovery in transplants with a major ABO mismatch and slightly lower hemoglobin levels as well. However, from about 9 months post HSCT, hemoglobin levels were unexpectedly higher post-transplant. Taken together, and since other studies report no difference between ABO matched and mismatched groups with respect to survival and relapse (17, 18), we see no evidence that ABO mismatch should be considered a major criterion in donor selection for pediatric patients.

Experiencing acute GvHD or a CMV or EBV reactivation did not impact long-term haematopoietic recovery, but CMV reactivations did have a transient negative impact on hemoglobin level for the first 6 months. This could be due to lingering direct effects from the CMV reactivation and associated treatment.

Since SCT aims for a lifelong cure and since overall survival has steadily improved it becomes increasingly important to study the long-term graft/stem cell function. Here we studied long term erythropoiesis and influencing factors during the first 3 years using novel modeling approaches and noted that patient specific factors appear more important for the final hemoglobin levels than donor factors. Modeling of long-term effects could help to re-evaluate choices made at the time of transplant regarding donor selection.

## Data Availability Statement

The datasets presented in this article are not readily available because of privacy concerns, dataset includes transplant dates, rare diagnoses and other information that could be used to identify patients. Requests to access the datasets should be directed to Erik G. J. von Asmuth, e.g.j.von_asmuth@lumc.nl.

## Ethics Statement

Ethical review and approval was not required for the study on human participants in accordance with the local legislation and institutional requirements. Written informed consent to participate in this study was provided by the participants' legal guardian/next of kin.

## Author Contributions

EA: study conception, acquisition of data, analysis and interpretation of data, and drafting of manuscript. HP: analysis and interpretation of data and critical revision. AM, MS, and AL: study conception and critical revision. All authors contributed to the article and approved the submitted version.

## Conflict of Interest

The authors declare that the research was conducted in the absence of any commercial or financial relationships that could be construed as a potential conflict of interest. The reviewer EM declared a past co-authorship with one of the authors AL to the handling editor.
