# Supplementary material for: Modeling Long-Term Erythropoietic Recovery After Allogeneic Stem Cell Transplants in Pediatric Patients
Source: Front Pediatr. 2020 Nov 30;8:584156. doi: 10.3389/fped.2020.584156 (PMC7734089; doi:10.3389/fped.2020.584156)
Supplement: Supplementary file 1 [file Data_Sheet_1.DOCX]

**Supplemental text 1: detailed statistical methodology**

Haemoglobin levels were modelled using a mixed model. Time was included in all models as a fixed effect using a B-spline with 5 knots (2 boundary knots at day 30 and day 1095 and 3 knots on the first quartile, median, and third quartile) and patient identity was included in all models as a random intercept effect to correct for multiple measurements. The measurements were inversely weighed according to proximity to other measurements from the same patient to avoid repeat measurements in close proximity having a strong effect on the model.

The models were created in R 3.6.0^15^. Models were fitted using lme4. Confidence intervals of predictions were calculated using a nonparametric block bootstrap using separate patients as blocks. Overall significances were determined by performing a Wald-test on the bootstrapped estimates. Model fit (R^2^) was calculated using leave-one-cluster-out cross validation.

To investigate the influence of different factors on recovery, we made separate models using the base model definition and primary disease, major ABO mismatch, stem cell source, patient age categorized as a separate category for every 3 years of age, and donor age categorized as 0-15 years, 15-25 years, 25-40 years, and 40+ years. All effects included interaction effects with time.

To investigate the influence of different factors on haemoglobin levels over 1-year post stem cell transplantation, an average of all haemoglobin values per patient between 1 and 3 years post stem cell transplantation was calculated, and an ANOVA was performed if there were more than 2 groups, or a T-test was performed if there were 2 groups. If the ANOVA was significant, we performed additional T-tests to investigate differences between groups.

*
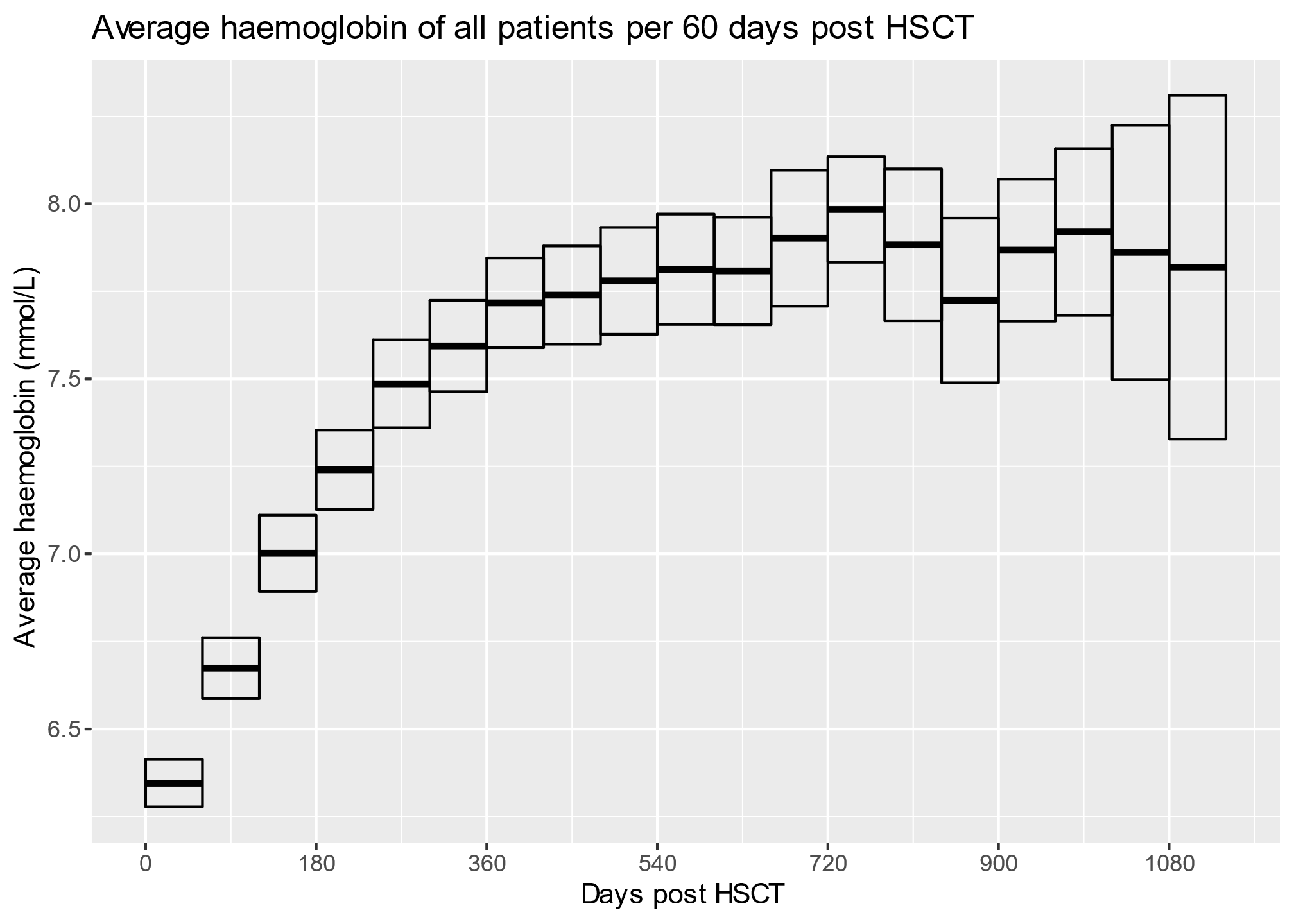
*

*Supplemental figure 1. Average haemoglobin level every 60 days, weighted for patient identity to give equal weight to all patients, with 95% confidence intervals.*


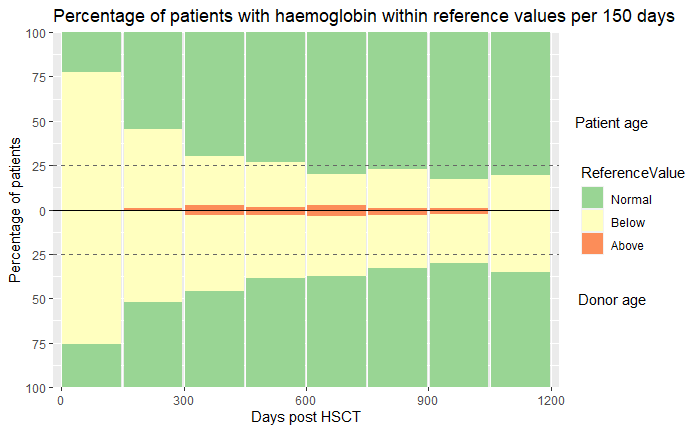


*Supplemental figure 2: Relative amount of patients having an average haemoglobin within or outside reference range based on patient or donor sex & age every 150 days. Reference values were calculated by using the age dependent upper and lower reference values described by Fulgoni et. al.^14^ for subjects over 1 year of age, and those of Takala et. al.^13^ for subjects under one year of age.*


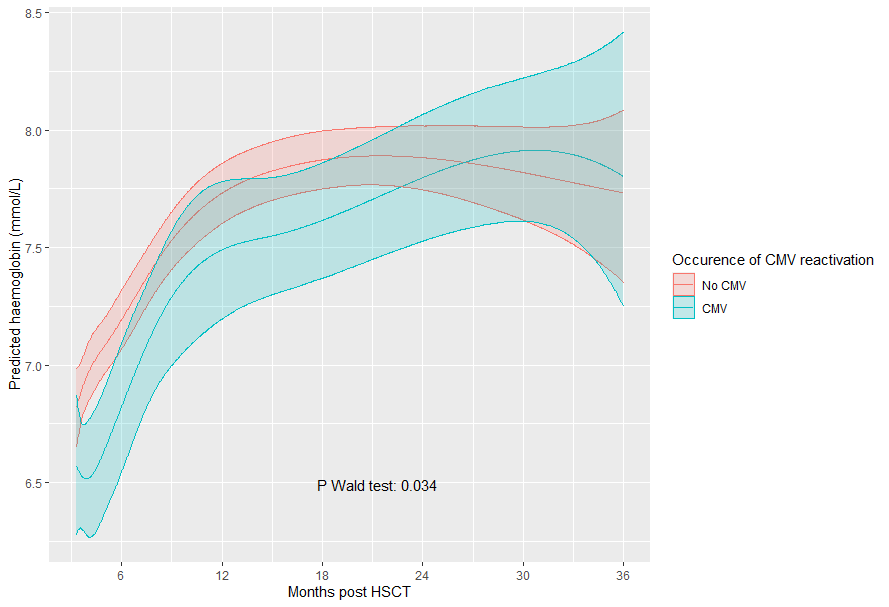


*Supplemental figure 3: Haemoglobin recovery split by having a CMV reactivation*

|  | R^2^ | COPD | R^2^ >1yr | COPD >1yr |
| --- | --- | --- | --- | --- |
| Only time | 0.259 | NA | 0.264 | NA |
| Diagnosis | 0.285 | 0.0355 | 0.328 | 0.0871 |
| ABO mismatch | 0.267 | 0.0103 | 0.284 | 0.0260 |
| Graft source | 0.266 | 0.0095 | 0.277 | 0.0175 |
| Patient age | 0.266 | 0.0088 | 0.279 | 0.0201 |
| Conditioning type | 0.258 | -0.0012 | 0.267 | 0.0036 |
| Donor age | 0.257 | -0.0028 | 0.239 | -0.0341 |

*Supplemental table 1: Predictive accuracy per model. COPD: coefficient of partial determination comparing the model to the model only including time*
